# Families of adolescents and young adults with complex chronic conditions: socioeconomic impact after ten years

**DOI:** 10.1590/0034-7167-2025-0057

**Published:** 2025-12-15

**Authors:** Fernanda Yeza Ferreira, Francisco Figueiredo, Mariane Caetano Sulino-Gonçalves, Regina Aparecida Garcia de Lima, Aline Cristiane Cavicchioli Okido

**Affiliations:** I Universidade Federal de São Carlos. São Carlos, São Paulo, Brazil; II Universidade de São Paulo. Ribeirão Preto, São Paulo, Brazil

**Keywords:** Adolescent, Young Adult, Chronic Disease, Family, Economic Status., Adolescente, Adulto Joven, Enfermedad Crónica, Familia, Estatus Económico.

## Abstract

**Objectives:**

to analyze the socioeconomic impact on families of adolescents and young adults with complex chronic conditions after ten years.

**Methods:**

we conducted a convergent parallel mixedmethods study. Eligible participants were mothers of adolescents and young adults with complex chronic conditions who had participated in a study conducted in 2012-2013. In the quantitative phase, the previously used socioeconomic questionnaire was reapplied. Descriptive statistics, the McNemar test, and the Wilcoxon test were used. In the qualitative phase, semistructured interviews identified convergences and divergences with the quantitative findings.

**Results:**

twenty-four mothers participated. No statistical difference was observed in household income. A decrease was found in families with homeownership and private health insurance. Challenges in formal employment, strategies to supplement income, and the high cost of rent were described.

**Conclusions:**

household income remained stable; however, consumption and access to goods decreased. Narratives corroborated the quantitative findings by describing the challenges experienced.

## RESUMEN

**Objetivos:**

analizar las repercusiones socioeconómicas en familias de adolescentes y adultos jóvenes con condiciones crónicas complejas tras diez años.

**Métodos:**

estudio de métodos mixtos paralelo convergente. Fueron elegibles madres de adolescentes y adultos jóvenes con condiciones crónicas complejas participantes en un estudio realizado em 20122013. En la fase cuantitativa se reaplicó el cuestionario socioeconómico usado entonces. Se emplearon estadística descriptiva, prueba de McNemar y prueba de Wilcoxon. En la fase cualitativa se realizaron entrevistas semiestructuradas para identificar convergencias y divergencias con los hallazgos cuantitativos.

**Resultados:**

participaron 24 madres. No se hallaron diferencias estadísticas en la renta familiar. Se observó reducción en familias con vivienda propia y seguro médico privado. Se retrataron desafíos del empleo formal, estrategias para complementar ingresos y el alto costo del alquiler.

**Conclusiones:**

la renta familiar se mantuvo, pero el consumo y acceso a bienes disminuyeron. Los testimonios reforzaron los hallazgos cuantitativos al describir los desafíos vividos.

## INTRODUCTION

Complex chronic conditions are defined as those that last at least 12 months (unless death occurs earlier), involve either multiple organs or systems or a single organ in severe form, and require specialized multiprofessional follow-up in tertiary care centers as well as the continuous use of medications and life-sustaining technologies such as tracheostomy and gastrostomy^(1,2)^.

Despite clinical vulnerability, advances in healthcare have improved the survival rates of children with complex chronic conditions over the years, and these children are now reaching adolescence and young adulthood^(3,4)^. In this context, the literature has focused on the challenges of self-management among adolescents with complex chronic conditions^(5)^ and on the transition from pediatric to adult care^(6,7)^, with few investigations addressing the socioeconomic impact.

Because complex chronic conditions are generally irreversible and represent a substantial economic burden for families, it is important to note that unfavorable social determinants may aggravate the complexity of the condition^(8)^. A Canadian study examining the effects of socioeconomic variables-such as distance to healthcare services, household income, sex, ethnicity, and caregiver educational attainment-on health outcomes in three groups of children with chronic diseases (cystic fibrosis, type 1 diabetes, and chronic kidney disease) identified household income as the main determinant of health. Children from families with an annual income below US$45,000 had significantly worse health conditions than those from families above this threshold. The association was moderate in the chronic kidney disease group (p = 0.03), but considerably stronger among children with type 1 diabetes (T1D) and cystic fibrosis (CF), both with high statistical significance (p < 0.01)^(9)^.

However, a systematic review examining the costs associated with the care of children with medical complexity found that 24 (89%) of the 27 studies analyzed assessed costs from the perspective of healthcare services, while only 3 (11%) investigated this issue from the family perspective^(10)^.

A scoping review analyzing cost indicators to measure the financial burden on families of children and adolescents with life-limiting conditions (such as cancer and cerebral palsy) highlighted another challenge: the different methodological approaches and variables used to assess families’ socioeconomic status. These inconsistencies limit the interpretation and comparability of research results, hindering the development and implementation of effective support policies^(11)^.

Based on these findings, the present study recontacted 102 mothers of children with complex chronic conditions who had participated in the doctoral dissertation of the last author. After ten years, they were asked: Have there been changes in families’ socioeconomic status? What were their experiences related to financial and work issues? In Brazil, a bibliographic search conducted in the main health research portals (BVS and PubMed) did not identify studies that longitudinally examined the socioeconomic impact on this population, underscoring the originality of this investigation.

## OBJECTIVES

To analyze the socioeconomic impact on families of adolescents and young adults with complex chronic conditions after ten years.

## METHODS

### Ethical aspects

The Research Ethics Committee of the Ribeirão Preto College of Nursing at the University of São Paulo approved the study, which adhered to the ethical principles outlined in Resolution No. 510/2016 of the Brazilian National Health Council.

### Study design, period, and setting

This is a convergent parallel mixed-methods study. In this design, quantitative and qualitative methods are combined to investigate complex phenomena, offering insights that would not be achieved with a single methodological approach^(12)^. In this study, data were equally weighted (QUAN+QUAL) and integrated in the Results section. The reporting of findings and manuscript preparation followed the Mixed Methods Appraisal Tool (MMAT)^(13)^, a framework increasingly used in nursing research to ensure methodological rigor in mixed-methods studies^(14)^.

Data collection occurred between October 2022 and August 2023 in a municipality in the northeastern region of the state of São Paulo. The contexts of data collection varied according to participants’ availability and preferences, including home visits, waiting rooms in healthcare facilities, and an online videoconferencing platform.

### Population and sample: inclusion and exclusion criteria

Eligible participants were mothers of adolescents and young adults with complex chronic conditions who had participated in the doctoral dissertation *Maternal experience in the care of technology-dependent children*. This dissertation, defended by the last author in 2013, aimed, among other objectives, to characterize technology-dependent children living in the municipality of Ribeirão Preto, São Paulo. For the present study, exclusion criteria were resolution of the clinical condition and death of the mother or child.

### Data collection and organization

For the recruitment process, an initial search was conducted in the Municipal Health Department’s electronic system (HygiaWeb) to verify the current clinical status of the adolescents/young adults. This search revealed that 44 (43.1%) of the 102 children who had participated in the 2012 study had died, and that two mothers and/or caregivers had also passed away.

The first strategy to contact the 56 remaining families was based on the addresses and telephone numbers provided in 2012; however, most of this information was outdated. The second strategy was to check scheduled medical, dental, or nursing appointments in healthcare facilities. Based on this information, the researchers attended the facilities on the scheduled dates to invite participants in person. Another strategy was to search social media using the full name of the mother and/or child.

Ultimately, 24 mothers and/or caregivers were located and agreed to participate. Of the 32 remaining families, 5 declined, 5 had resolved the child’s health condition, and 22 were excluded after five unsuccessful attempts at contact.

Data collection took place at dates, times, and locations chosen by the participants and lasted approximately 40 minutes. First, the same socioeconomic questionnaire used ten years earlier was reapplied, covering the following variables: household income; government benefits; housing type; household size; number of rooms; access to running water, sewage system, and electricity; and type of healthcare coverage.

Semi-structured interviews were conducted immediately after completion of the questionnaire with a purposive subsample of mothers. Continuation generally depended on participant availability; 12 mothers agreed to share their experiences. The core prompt of the semi structured interview was: *Tell me about your experiences related to financial and work issues over the past ten years*.

### Data Analyses

Quantitative data were organized and analyzed as follows: responses from the socioeconomic questionnaire were first entered into an Excel spreadsheet and coded. Data collected in 2012-2013 were then incorporated to allow comparison with the current data and to identify socioeconomic impacts over the ten-year period.

The database was exported to SAS for Windows, version 9.4 (Statistical Analysis System), and analyses were performed with the support of a statistician. Data were initially described using absolute and relative frequencies (qualitative variables) and measures of central tendency and dispersion, including mean, standard deviation, minimum, median, and maximum (quantitative variables).

To ensure accurate comparisons across years, household income was updated by multiplying the reported amount by the accumulated IPCA factor for the period (1.71227860, considering the index from December 2012 to December 2021). The calculation was performed using the Citizen’s Calculator provided by the Central Bank of Brazil, available online free of charge. The IPCA was chosen because it is the official inflation index used by the Central Bank of Brazil.

Comparisons of categorical variables were conducted with the McNemar test. Comparisons of “number of rooms” and “household size” used a regression model with a negative binomial distribution, a log link function, and repeated measures, given that these outcomes are discrete rather than continuous quantitative variables. Comparisons involving minimum wage and household income were analyzed with the Wilcoxon signed-rank test for paired samples.

In the qualitative phase, interviews were audio-recorded, fully transcribed, and analyzed by the lead researcher and coauthors without software support. A careful reading of the transcripts was performed to identify convergences and divergences between qualitative and quantitative findings.

## RESULTS

As noted earlier, 24 mothers of adolescents and young adults with complex chronic conditions, aged 12 to 19 years, participated in the quantitative phase. In the qualitative phase, 12 mothers shared their experiences.

One of the socioeconomic aspects compared in this study was housing type. As shown in Table 1, there was a 19.2% decrease in homeownership, with a corresponding increase in families living in provided housing.

**Table 1:** Distribution of housing type according to year (2012 and 2022), Ribeirão Preto, São Paulo, Brazil, 2024

Housing type	2012	2022
Owned	14 (60.9%)	10 (41.6%)
Rented	6 (26.1%)	7 (29.2%)
Provided	3 (13%)	7 (29.2%)
Missings	1	0

This numerical finding was corroborated by qualitative accounts:

*I live in the back house, and my grandmother is in the front house.* (M5, 46 years, mother of a 15-year-old adolescent who required bowel irrigation and intermittent bladder catheterization)

*I moved in to take care of my grandmother; after she passed away, I stayed in the house.* (M8, 44 years, mother of a 15-year-old adolescent who required diapers, a wheelchair, and assisted feeding)

Leaving one’s own home does not always mean a worsening of the family’s living conditions. In this regard, the statement below conveys a positive aspect of moving into a house provided by the daughter.

*This house I’m living in was bought by my oldest daughter* [from my first marriage]*. At the time of the first interview* [2012]*, I lived in the community. It was tough, with sewage and garbage at my doorstep.* (M7, 46 years, mother of a 19-year-old young adult who used tracheostomy and gastrostomy)

The number of families renting a home remained nearly the same over the past ten years. However, the narratives highlight how burdensome this monthly expense is:

*It would be a great help if we had our own house, because it’s hard: you pay, and before you know it, the rent is due again. But even though we don’t own a house, we still have stability at home. We buy things little by little, tighten the budget here and there, but we get by.* (M2, 46 years, mother of a 22-year-old young adult who required intermittent bladder catheterization)

*Now I found this cheaper little house. It’s working out.* (M9, mother of a 14-year-old adolescent who used tracheostomy and gastrostomy)

When comparing household income between 2012 and 2022, no statistically significant difference was found in “household income in reais” or “household income in minimum wages”, as shown in Table 2. Furthermore, the percentage of families receiving government benefits also remained similar, with no statistical difference. These results indicate that, overall, the families remained in class D (one to three minimum wages) and class E (up to one minimum wage), according to the IBGE classification.

**Table 2:** Distribution of “household income” and “government benefit” according to year (2012 and 2022), Ribeirão Preto, São Paulo, Brazil, 2024

	2012	2022	*p* value
Household income (BRL)			0.33^**^
Mean (SD)	2.817 (2.104)	3.151 (2.464)	
Median (Min.-Max.)	2.311 (513-10.273)	2.500 (1.280-12.000)	
Household income (minimum wage)			0.91^**^
Mean (SD)	2.64 (1.97)	2.6 (2.03)	
Median (Min.-Max.)	2.17 (0.48-9.63)	2.06 (1.06-9.9)	
Government benefit			0.32^*^
Yes	15 (65.22%)	18 (75%)	
No	8 (34.78%)	6 (25%)	
Missings	1	0	

Min. - minimum; Max. - maximum; SD - standard deviation ;

* Wilcoxon signed-rank test for paired samples;

** McNemar test.

One possible explanation for the persistence of limited purchasing power among these families over the years is the constant challenge faced by mothers in entering formal employment. The need for continuous supervision and care generally remained, even as their children reached adolescence or young adulthood.

*I always wanted to have a formal job, but I never could. How could I work formally like this? Do you think an employer would accept me saying, “Look, I have a daughter with special healthcare needs, and I have to rush home. Something happened, I need to go”? Things keep coming up all the time.* (M2, 46 years, mother of a 22-year-old young adult who required intermittent bladder catheterization)

*I was never able to work after he was born. I used to work as a cook and nanny. I traveled more than I stayed in Ribeirão. I worked for a family that traveled frequently to Minas, and I often accompanied them. I worked there for eight years. Then I got married, got pregnant, and left* […]. *My job today is my son.* (M6, 50 years, mother of a 15-year-old adolescent who used tracheostomy and gastrostomy)

*I used to work, but I had to stop because I couldn’t manage anymore. I wasn’t sleeping. At night, he had seizures, he stayed awake, and I had to stay awake to watch him so nothing serious would happen.* (M10, 52 years, mother of a 17-year-old adolescent who used tracheostomy and gastrostomy)

*I devoted my life to caring for him. From the moment I took him in, he needed 24 hour care. He was on oxygen, fed through a tube. Even when sleeping, I had to stay alert, afraid that something might happen.* (M12, 64 years, adoptive mother of a 15-year-old adolescent with reduced mobility and ventriculoperitoneal shunt)

In an effort to contribute to household expenses, some mothers sought informal work.

*I’m always picking up odd jobs: selling lingerie, doing manicures, ironing clothes-always to be able to help a little more.* (M5, 46 years, mother of a 15-year-old adolescent who required bowel irrigation and intermittent bladder catheterization)

*Whenever there was a craft fair, I took my handicrafts, sold them, and bought what was needed for the house.* (M2, 46 years, mother of a 22-year-old young adult who required intermittent bladder catheterization)

In addition to household income, other socioeconomic variables considered by the IBGE for social class classification were also investigated in this study, namely household size, housing dimensions, and access to running water, sewage system, and electricity. As shown in Table 3, there was an estimated 18% increase in household size after ten years (p = 0.04).

**Table 3:** Distribution of “household size”, “number of rooms”, and “presence of running water, sewage system, and electricity” according to year (2012 and 2022), Ribeirão Preto, São Paulo, Brazil, 2024

	2012	2022	Effect (95% CI)	*p* value^***^
Household size				
Mean (SD)	2.78 (1.17)	3.29 (0.69)	1.18 (1.01;1.39)	**0.04**
Median (Min.-Max.)	2 (1-5)	3 (2-4)		
Number of rooms			0.88 (0.77;1.01)	0.07
Mean (SD)	5.35 (1.56)	4.71 (1.16)		
Median (Min.-Max.)	6 (2-8)	4.5 (3-8)		
Running water, sewage system, and electricity				
Yes	23(96%)	24(100%)		
No	01(4%)	-		

Min. - minimum; Max. - maximum; SD - standard deviation ;

*** Negative binomial regression model with log link function considering repeated measures.

The increase in household size may be explained by more people moving into the same residence, as some families relocated to provided housing. Conversely, over the past decade, difficulties in marital relationships led to partners leaving the home, as illustrated by Mother 7.

*Her father left me because he couldn’t handle it. All he cared about was drinking, and there were constant arguments. So, when you’re with someone and you can never count on him, it’s better to be alone.* (M7, 46 years, mother of a 19-year-old young adult who used tracheostomy and gastrostomy)

Another socioeconomic variable analyzed was the type of healthcare coverage. As shown in Table 4, there was a 50% decrease in families with both SUS and private health insurance (mixed coverage), with a corresponding increase in families relying exclusively on the Brazilian Unified Health System (SUS).

**Table 4:** Distribution of type of healthcare coverage according to year (2012 and 2022), Ribeirão Preto, São Paulo, Brazil, 2024

Type of healthcare coverage	2012	2022
Brazilian Unified Health System (SUS)	13 (54.2%)	17 (70.8%)
Private health insurance	1 (4.2%)	2 (8.4%)
Mixed coverage	10 (41.6%)	5 (20.8%)

This scenario proved unfavorable, as dissatisfaction with public services-particularly Primary Health Care-was underscored in qualitative accounts. In these cases, private health insurance was described as complementary, as illustrated by Mother 6.

*Because my son is invisible. I’ve been to the health center countless times. My son is invisible to the SAD* [Home Care Service]*; it’s as if he doesn’t exist. We have a doctor who comes every month. I used to have it* [tracheostomy tube] *changed at the University Hospital* (HC)*. When the pandemic began, we were afraid to go there, so we asked the insurance company to send a doctor to our home every month. She now comes through the insurance home care service.* (M6, 50 years, mother of a 15-year-old adolescent who used tracheostomy and gastrostomy)

## DISCUSSION

According to the results, no statistically significant difference was found in household income, whether in reais or in minimum wages, over the past ten years, with the families remaining in the lowest social classes according to the IBGE classification. In addition, there was an 18% increase in household size, which negatively affected families’ purchasing power as the per capita income decreased.

Although type 1 diabetes mellitus (T1D) is not considered a complex chronic condition, this finding is consistent with a study conducted in the state of Paraíba with 81 caregivers of children and adolescents with T1D, in which 40.7% (n = 33) lived on a household income below one minimum wage and 58% (n = 47) lived in households with three or four additional people besides the child or adolescent^(15)^.

An investigation by researchers at the Oswaldo Cruz Foundation (FIOCRUZ) that analyzed the challenges of hospital discharge and clinical stability in children with complex chronic conditions highlighted the importance of addressing social issues. The authors found that most families had an income below one-quarter of the per capita minimum wage, with the Continuous Cash Benefit (BPC) as their primary source of income. This finding underscores a situation of social vulnerability that hinders the hospital discharge process^(16)^.

By contrast, a U.S. study with 601 parents of individuals aged 0 to 28 years in palliative care sought to determine the relationship between financial hardship and parents’ psychological distress. Approximately 13.2% (n = 79) reported household incomes below US$20,000, which is under the national poverty line of US$21,960 for a family of three^(17)^, a percentage lower than that observed in the present study.

Additionally, the authors applied a subjective measure to assess financial hardship, asking participants about their level of difficulty in paying bills over the previous six months. Sixty-five percent reported some degree of financial difficulty. This finding supported the hypothesis that financial challenges may occur across all income levels, suggesting that the perception of financial hardship extends beyond household income^(17)^.

In the present study, among the 24 participating families, 60.9% owned their homes in 2012, a percentage that dropped to 41.6% in 2022. By comparison, a Canadian study investigating the impact of parental caregiving on 15 families of children and young people with medical complexity found that about 70% owned their homes. However, participants also reported challenges related to the high costs of adapting homes and vehicles to improve accessibility for their children^(18)^.

Another relevant aspect is the sharp increase in the prices of food, rent, and other basic expenses, which resulted in loss of purchasing power. According to the Inter-Union Department of Statistics and Socioeconomic Studies (DIEESE), the cost of the basic food basket rose from R$304.90 in December 2012 to R$791.29 in December 2022-an increase of about 50% over ten years (reference: São Paulo capital)^(19)^.

Overall, studies on the financial impact of families with members who have chronic health conditions distinguish between direct and indirect costs. Direct costs include expenses such as purchasing medications and supplies, paying for private health insurance, and transportation to hospitals^(11)^. In the present study, a reduction in private health insurance coverage was observed over the ten years, representing another negative socioeconomic impact. Indirect costs are associated with changes in productivity and employment, such as the need for caregivers to reduce or abandon work, leading to loss of income^(11)^.

From this perspective, the results reaffirm the ongoing challenge faced by mothers as caregivers in entering formal employment. This issue was emphasized in a study that examined the impact of cerebral palsy on health and finances^(20)^. According to the researchers, the economic impact of income loss among informal caregivers (generally mothers) is significant, as the direct medical care costs for children with cerebral palsy aged 1 to 4 years were approximately US$11,700, compared with about US$600 for children without the condition.

The difficulty of entering the labor market, combined with the burden of caregiving demands, is consistently highlighted in the literature. A U.S. study found that caregivers had to reorganize their work routines to meet the needs of children with medical complexity: 36% left their jobs, 66% remained employed with reduced hours, and 46.2% avoided changing jobs to keep their health insurance^(21)^. In a national study with parents of children and adolescents with special healthcare needs, the exhausting routine, combined with the lack of support and the intense demand for care, led the primary caregiver-usually the mother-to abandon employment^(22)^.

In another study conducted in Hong Kong on the socioeconomic burden of rare diseases, direct and indirect costs accounted for more than 60% of the total cost. However, older age of the person with the rare disease (p < 0.0001) and longer time since diagnosis (p = 0.026) were negatively associated with the mean annual total cost^(23)^.

It should also be noted that data collection took place between 2022 and 2023, a period that coincided with the end of the Public Health Emergency of International Concern (PHEIC), officially declared by the World Health Organization on May 5, 2023. This context had considerable socioeconomic impacts on the population. A review study investigating the effects of social determinants during the COVID-19 pandemic in the pediatric population reported increases in housing and food insecurity, along with a global rise in poverty rates^(24)^. Corroborating these findings, a qualitative study with 27 individuals who contracted COVID-19 identified financial changes as one of the main negative impacts of the pandemic, particularly unemployment, inability to work, and treatment-related costs-factors that most severely affected families already in situations of socioeconomic vulnerability^(25)^.

Finally, this investigation aligns with the 2030 Agenda for Sustainable Development^(26)^, as it explored the interrelationship between health and well-being, poverty and hunger eradication, and reduction of inequalities. This approach is particularly relevant in light of a scoping review that sought to identify and analyze the scientific literature on the relationship between nursing and the Sustainable Development Goals (SDGs). The review found that nursing practice remains predominantly focused on SDG 3 - Good Health and Well-Being, as it is considered the most directly linked to professional nursing practice^(27)^. In this regard, researchers have argued that health should be addressed through a broader lens that goes beyond traditional individualand disease-centered models, incorporating social, economic, and environmental determinants^(28,29)^.

### Study limitations

A limitation of this study is the small number of participants, corresponding to only 20% of those who took part in the research conducted ten years ago. However, the restricted sample does not compromise the validity of the findings and underscores the importance of conducting new longitudinal studies with a larger number of families. Future research should also, whenever possible, include the perspectives of adolescents and young adults with complex chronic conditions.

### Contributions to the field

This study advances nursing knowledge through its originality in integrating quantitative and qualitative results to explore the socioeconomic repercussions experienced by mothers of adolescents and young adults with complex chronic conditions. The findings also have the potential to inform the planning of intersectoral actions aimed at promoting comprehensive care, particularly for families living in poverty.

Accordingly, the development and implementation of public policies that expand support for caregiving families are recommended. Such policies include access to income transfer programs linked to the care of people with chronic conditions, prioritization in housing programs, provision of vocational training opportunities, and adoption of flexible work arrangements, including specific leave entitlements for continuous treatment or frequent hospitalizations of children.

## Data Availability

The research data are available within the article.

## References

[1] Menezes LAD, Carvalho KM, Gomes MADSM, Carvalho MSND. (2023). Análise da produção científica nacional das condições crônicas complexas em pediatria. Saúde Debate.

[2] Leyenaar JK, Schaefer AP, Freyleue SD, Austin AM, Simon TD, Van Cleave J (2022). Prevalence of children with medical complexity and associations with health care utilization and in-hospital mortality. JAMA Pediatr.

[3] Nightingale R, McHugh G, Kirk S, Swallow V. (2019). Supporting children and young people to assume responsibility from their parents for the self-management of their long-term condition: an integrative review. Child Care Health Dev.

[4] Pitts L, Patrician PA, Landier W, Kazmerski T, Fleming L, Ivankova N (2024). Parental entrustment of healthcare responsibilities to youth with chronic conditions: a concept analysis. J Pediatr Nurs.

[5] Gauci J, Bloomfield J, Lawn S, Towns S, Steinbeck K. (2021). Effectiveness of self-management programmes for adolescents with a chronic illness: a systematic review. J Adv Nurs.

[6] Toulany A. (2022). Quality indicators for youth transitioning to adult care: a systematic review. Pediatrics.

[7] Bailey K, Lee S, Los Reyes T, Lo L, Cleverley K, Pidduck J. (2022). Quality indicators for youth transitioning to adult care: a systematic review. Pediatrics.

[8] Lozano P, Houtrow A. (2018). Supporting self-management in children and adolescents with complex chronic conditions. Pediatrics.

[9] Seear M, Amed S, Dionne J, Yang C, Tourigny K, De Mello A (2019). In support of point-of-care social needs screening: the effects of five social determinants on the health of children with chronic diseases in British Columbia. Paediatr Child Health.

[10] Sidra M, Sebastianski M, Ohinmaa A, Rahman S. (2024). Reported costs of children with medical complexity-A systematic review. J Child Health Care.

[11] Mitterer S, Zimmermann K, Bergsträsser E, Simon M, Gerber AK, Fink G. (2021). Measuring financial burden in families of children living with life-limiting conditions: a scoping review of cost indicators and outcome measures. Value Health.

[12] Creswell JW, Plano Clark VL. (2013). Lopes MF, tradutor.

[13] Mixed Methods Appraisal Tool (MMAT) (2018). Version 2018.

[14] Oliveira JLC, Magalhães AMMD, Matsuda LM, Santos JLGD, Souto RQ, Riboldi CDO (2021). Mixed methods appraisal tool: Strengthening the methodological rigor of mixed methods research studies in nursing. Texto Contexto Enferm.

[15] Cavalcante MEPL, Ramalho ELR, Pessoa MSA, Oliveira RC, Sparapani VC, Nascimento LC (2023). Perfil social e clínico de crianças e adolescentes com diabetes mellitus tipo 1. Rev Enferm UFSM.

[16] Barbosa FDO. (2022). Crianças com condições crônicas complexas de saúde moradoras de uma pediatria: por que é tão difícil desospitalizar?.

[17] Boyden JY, Hill DL, Nye RT, Bona K, Johnston EE, Hinds P (2022). Pediatric palliative care parents’ distress, financial difficulty, and child symptoms. J Pain Symptom Manage.

[18] Teicher J, Moore C, Esser K, Weiser N, Arje D, Cohen E (2023). The experience of parental caregiving for children with medical complexity. Clin Pediatr.

[19] Departamento Intersindical de Estatística e Estudos Socioeconômicos (DIEESE) (2022). Cesta básica - evolução mensal: São Paulo.

[20] Amankwah N, Oskoui M, Garner R, Bancej C, Manuel DG, Wall R (2020). Original quantitative research-cerebral palsy in Canada, 2011-2031: results of a microsimulation modelling study of epidemiological and cost impacts. Health Promot Chronic Dis Prev Can.

[21] Lawrence PR, Faulkner MS, Spratling R. (2025). Social support and family resources influence workload and capacity in parents of children with medical complexity. J Pediatr Nurs.

[22] Nonose ERDS, Silva RMMD, Neves ET, Mello DFD, Zilly A, Okido ACC (2024). Saúde mental de pais de crianças e adolescentes que necessitam de atenção especial à saúde. Rev Bras Enferm.

[23] Chung CC, Ng NY, Ng YN, Lui AC, Fung JL, Chan MC (2023). Socio-economic costs of rare diseases and the risk of financial hardship: a cross-sectional study. Lancet Reg Health West Pac.

[24] Abrams EM, Greenhawt M, Shaker M, Pinto AD, Sinha I, Singer A. (2022). The COVID-19 pandemic: adverse effects on the social determinants of health in children and families. Ann Allergy Asthma Immunol.

[25] Barreto MDS, Marques FRDM, Gallo AM, Garcia-Vivar C, Carreira L, Salci MA. (2023). Conquistando um novo equilíbrio: um estudo qualitativo de como a vida familiar foi afetada pela COVID-19. Rev Latino-Am Enfermagem.

[26] Organização das Nações Unidas (ONU) (2015). Agenda 2030 para o Desenvolvimento Sustentável.

[27] Fields L, Perkiss S, Dean BA, Moroney T. (2021). Nursing and the Sustainable Development Goals: a scoping review. J Nurs Scholarsh.

[28] Rosa WE, Dossey BM, Watson J, Beck DM, Upvall MJ. (2019). The United Nations Sustainable Development Goals: the ethic and ethos of holistic nursing. J Holist Nurs.

[29] Kitt-Lewis E, Adam M, Buckland P, Clark D, Hockenberry K, Jankura D (2020). Creating a generation of sustainable nurses: sustainability efforts in nursing education. Nurs Clin North Am.

