# Supplementary material for: Families of adolescents and young adults with complex chronic conditions: socioeconomic impact after ten years
Source: Rev Bras Enferm. 2025 Dec 15;78(5):e20250057. doi: 10.1590/0034-7167-2025-0057 (PMC12707852; doi:10.1590/0034-7167-2025-0057)
Supplement: Translation

# Translation: Famílias de adolescentes e adultos jovens com condições crônicas complexas: repercussões socioeconômicas após dez anos

**DOI:** 10.1590/0034-7167-2025-0057pt

**Authors:** Fernanda Yeza Ferreira, Francisco Figueiredo, Mariane Caetano Sulino-Gonçalves, Regina Aparecida Garcia de Lima, Aline Cristiane Cavicchioli Okido

**Affiliations:** I Universidade Federal de São Carlos. São Carlos, São Paulo, Brasil; II Universidade de São Paulo. Ribeirão Preto, São Paulo, Brasil

**Keywords:** Adolescente, Adulto Jovem, Doença Crônica, Família, *Status* Econômico.

## Abstract

**Objetivos:**

analisar as repercussões socioeconômicas nas famílias de adolescentes e jovens adultos com condições crônicas complexas após dez anos.

**Métodos:**

estudo de métodos mistos do tipo paralelo convergente. Foram elegíveis mães de adolescentes e adultos jovens com condições crônicas complexas participantes de estudo realizado em 2012-2013. Na etapa quantitativa, reaplicou-se o questionário socioeconômico utilizado naquela época. Utilizaram-se estatística descritiva, Teste de McNemar e Teste de Wilcoxon nas comparações. Na etapa qualitativa, conduziram-se entrevistas semiestruturadas para identificar convergências e divergências com os achados quantitativos.

**Resultados:**

participaram 24 mães. Não houve diferença estatística para renda familiar. Observou-se redução no número de famílias com casa própria e convênio médico. Desafios do trabalho formal, estratégias para complementar a renda e custo elevado do aluguel foram retratados.

**Conclusões:**

a renda familiar manteve-se, todavia o consumo e acesso a bens reduziram. Os depoimentos reforçaram os achados quantitativos ao descreverem os desafios vivenciados.

## INTRODUÇÃO

As condições crônicas complexas são caracterizadas por uma duração mínima de 12 meses (salvo se a morte ocorrer antes desse período), envolvem múltiplos órgãos ou sistemas, ou um único órgão de forma severa, requerem acompanhamento multiprofissional especializado em centros de atenção terciária e demandam o uso contínuo de medicações e tecnologias para manutenção da vida, como traqueostomia e gastrostomia, por exemplo^(1,2)^.

Apesar da fragilidade clínica, os avanços nos cuidados de saúde têm contribuído para a melhoria nas taxas de sobrevivência de crianças com condições crônicas complexas ao longo dos anos, e essas crianças estão agora tornando-se adolescentes e jovens adultos^(3,4)^. Nesse contexto, a literatura tem se concentrado nos desafios do autogerenciamento de adolescentes com condições crônicas complexas^(5)^ e na transição do cuidado pediátrico para os serviços adultos^(6,7)^, com poucas investigações sobre as repercussões socioeconômicas.

Considerando que as condições crônicas complexas geralmente são irreversíveis e representam um alto custo econômico para as famílias, é importante ressaltar que determinantes sociais desfavoráveis podem agravar a complexidade da condição^(8)^. Nesse sentido, um estudo canadense que investigou os efeitos de variáveis socioeconômicas - como distância até os serviços de saúde, renda familiar, gênero, etnia e escolaridade do cuidador - sobre os desfechos de saúde em três grupos de crianças com doenças crônicas (fibrose cística, diabetes tipo 1 e doença renal crônica) identificou a renda familiar como o principal determinante da saúde. Crianças provenientes de famílias com renda anual inferior a US$ 45 mil apresentaram condições de saúde significativamente mais desfavoráveis em comparação àquelas pertencentes a famílias com renda superior a esse valor de referência. A associação foi moderada no grupo com doença renal crônica (p = 0,03), porém substancialmente mais forte entre as crianças com diabetes tipo 1 (DM1) e fibrose cística (FC), em ambos os casos com significância estatística elevada (p < 0,01)^(9)^.

Contudo, uma revisão sistemática que analisou os custos associados ao cuidado de crianças com complexidade médica revelou que 24 (89%) dos 27 estudos analisados abordaram os custos sob a perspectiva dos serviços de saúde, enquanto apenas 3 (11%) investigaram essa temática sob a perspectiva familiar^(10)^.

De acordo com uma revisão de escopo que analisou indicadores de custo para medir o ônus financeiro das famílias de crianças e adolescentes com condições limitantes da vida (como câncer e paralisia cerebral), outra problemática envolve as diferentes abordagens metodológicas e variáveis utilizadas para medir a condição socioeconômica das famílias, o que limita a interpretação e comparabilidade dos resultados de pesquisa, além de dificultar o desenvolvimento e a implementação de políticas de apoio eficazes^(11)^.

Considerando o exposto, o presente estudo buscou reaproximar-se das 102 mães de crianças com condições crônicas complexas que participaram da tese de doutorado da última autora. O intuito foi que, após dez anos, elas respondessem aos seguintes questionamentos: Houve alterações na condição socioeconômica das famílias? Quais foram as experiências relacionadas às questões financeiras e de trabalho? Cabe destacar que, no Brasil, com base em uma busca bibliográfica realizada nos principais portais de pesquisa em saúde (BVS e PubMed), não foram identificados estudos que analisassem longitudinalmente as repercussões socioeconômicas nessa população, o que reforça a originalidade da presente investigação.

## OBJETIVOS

Analisar as repercussões socioeconômicas nas famílias de adolescentes e jovens adultos com condições crônicas complexas após dez anos.

## MÉTODOS

### Aspectos éticos

O Comitê de Ética em Pesquisa da Escola de Enfermagem de Ribeirão Preto da Universidade de São Paulo analisou e aprovou o projeto de pesquisa, que atendeu aos princípios éticos exigidos na Resolução nº 510/2016 do Conselho Nacional de Saúde.

### Desenho, período e local do estudo

Trata-se de um estudo de métodos mistos, utilizando a estratégia paralelo convergente. Nesse tipo de estudo, ocorre o uso associado dos métodos quantitativo e qualitativo como uma alternativa para estudar fenômenos complexos, pois promove um entendimento que não seria alcançado com uma única abordagem metodológica^(12)^. No presente estudo, os dados tiveram igual atribuição de peso (QUAN+QUAL) e foram integrados na seção dos resultados. A apresentação dos achados e a redação deste manuscrito seguiram as diretrizes do *Mixed Methods Appraisal Tool* ^(13)^, ferramenta que vem sendo utilizada por pesquisadores da área de enfermagem que buscam rigor metodológico ao planejar e conduzir estudos de métodos mistos^(14)^.

A coleta de dados ocorreu entre outubro de 2022 e agosto de 2023, em um município localizado na região nordeste do estado de São Paulo. Todavia, os contextos de produção do material empírico foram diversos, pois dependeram da escolha e disponibilidade das participantes. Nessa direção, a coleta de dados foi realizada em domicílios, em salas de espera das unidades de saúde e, também, via plataforma on-line de videochamadas.

### População e amostra: critérios de inclusão e exclusão

Foram elegíveis mães de adolescentes e adultos jovens com condições crônicas complexas que fizeram parte da tese de doutorado intitulada “Experiência materna no cuidado do filho dependente de tecnologia”. Essa tese foi defendida pela última autora em 2013 e tinha como um dos seus objetivos caracterizar as crianças dependentes de tecnologia residentes no município de Ribeirão Preto - SP. Para o presente estudo, os critérios de exclusão foram a resolução do quadro clínico e o óbito da mãe ou do(a) filho(a).

### Coleta e organização dos dados

Quanto ao processo de recrutamento, inicialmente foi realizada uma busca no sistema informatizado da Secretaria Municipal de Saúde (HygiaWeb) a fim de certificar-se da condição clínica atual do adolescente/adulto jovem. Com isso, identificou-se que 44 (43,1%) das 102 crianças participantes do estudo em 2012 foram a óbito. Também, verificou-se que duas mães e/ou responsáveis faleceram.

A primeira estratégia adotada para o contato inicial com as 56 famílias não excluídas inicialmente foi a busca com base nas informações de endereço e contato telefônico informados em 2012, todavia observou-se que a maioria dos dados estavam desatualizados. A segunda estratégia foi consultar os agendamentos nos serviços de saúde, para atendimento médico, odontológico ou de enfermagem. Com essa informação, as pesquisadoras compareceram nas unidades no dia e hora agendados para realizar o convite pessoalmente. Outra estratégia adotada foi a busca nas redes sociais utilizando o nome completo da mãe e/ou do(a) filho(a).

Ao final, foram localizadas e aceitaram participar 24 mães e/ou responsáveis. Das 32 restantes, 5 se recusaram a participar da pesquisa, 5 tiveram a condição de saúde do filho(a) solucionada, e 22 foram descontinuadas após cinco tentativas de localização ou contato sem sucesso.

A coleta de dados aconteceu em dia, horário e local definido pelas participantes e tiveram duração aproximada de 40 minutos. Inicialmente, aplicava-se o mesmo instrumento de caracterização socioeconômica utilizado há dez anos, contemplando as seguintes variáveis: renda familiar, benefício do governo, tipo de moradia, número de moradores, número de cômodos, presença de água encanada, rede de esgoto e energia elétrica e tipo de atendimento à saúde.

A entrevista semiestruturada foi efetuada imediatamente após o término da aplicação do instrumento e foi conduzida com uma parcela intencional das mães. Em geral, o critério adotado para dar continuidade era a disponibilidade da participante; assim, 12 mães aceitaram narrar suas experiências. O enunciado norteador da entrevista semiestruturada foi: Conte-me sobre suas vivências com relação às questões financeiras e de trabalho nos últimos dez anos.

### Análise dos dados

Os dados da etapa quantitativa foram organizados e analisados da seguinte forma: primeiramente, as respostas do instrumento de caracterização socioeconômica foram lançadas numa planilha Excel e codificadas. Posteriormente, foram incluídos os dados coletados em 2012-2013 a fim de possibilitar a comparação com os dados atuais e identificar possíveis repercussões socioeconômicas nos últimos dez anos.

Em seguida, o banco foi exportado para o *The SAS System for Windows* (*Statistical Analysis System*) - versão 9.4, no qual foram realizadas as análises com assessoria de um profissional estatístico. Inicialmente os dados foram descritos com uso de frequências absolutas e percentuais (variáveis qualitativas) e medidas de centralidade e de dispersão como média, desvio-padrão, mínimo, mediana e máximo (variáveis quantitativas).

Para permitir a real comparação entre os anos, a atualização da renda familiar foi obtida multiplicando-se o valor a ser corrigido pelo fator acumulado do IPCA no período (de 1,71227860 considerando o índice do período de 12/2012 a 12/2021). O cálculo foi realizado utilizando a calculadora do cidadão do banco central, disponível gratuitamente na internet. Optou-se pelo uso do IPCA para atualização da renda por se tratar do índice oficial de inflação utilizado pelo Banco Central do Brasil.

As comparações das variáveis tratadas como categóricas foram analisadas por meio do teste de McNemar. Para comparar as variáveis “número de cômodos” e “número de moradores”, utilizou se o modelo de regressão com distribuição binomial-negativa com função de ligação logarítmica e considerando medidas repetidas, dado que esses desfechos são variáveis quantitativas discretas e não contínuas. As comparações envolvendo os salários mínimos e renda familiar foram analisadas por meio do teste de Wilcoxon considerando amostras pareadas.

Na etapa qualitativa, as entrevistas audiogravadas foram transcritas na íntegra e analisadas pela pesquisadora principal e demais autoras que contribuíram no estudo, sem uso de software. Assim, procedeu-se à leitura cuidadosa das entrevistas, visando identificar convergências e divergências entre os achados qualitativos e quantitativos.

## RESULTADOS

Conforme descrito anteriormente, participaram da etapa quantitativa 24 mães de adolescentes e adultos jovens com condições crônicas complexas, com idade entre 12 e 19 anos. Na etapa qualitativa, 12 mães relataram suas experiências.

Um dos aspectos socioeconômicos comparados no presente estudo diz respeito ao tipo de moradia. De acordo com a Tabela 1, houve redução de 19,2% entre aqueles que tinham casa própria, com consequente aumento no número de famílias que passaram a residir em casas cedidas.

**Tabela 1:** Distribuição do tipo de moradia segundo o ano (2012 e 2022), Ribeirão Preto, São Paulo, Brasil, 2024

Tipo de moradia	2012	2022
Própria	14 (60,9%)	10 (41,6%)
Alugada	6 (26,1%)	7 (29,2%)
Cedida	3 (13%)	7 (29,2%)
*Missings*	1	0

*Missings - dados ausentes.*

Tal resultado numérico é corroborado por alguns relatos qualitativos:

*Moro aqui no fundo, minha vó na casa da frente.* (M5, 46 anos, mãe de adolescente de 15 anos que realizava lavagem intestinal e cateterismo vesical intermitente)

*Eu fui para cuidar da minha vó, depois ela faleceu e ainda continuei na casa.* (M8, 44 anos, mãe de adolescente de 15 anos que requeria uso de fraldas, cadeira de rodas e alimentação assistida)

Deixar de morar em local próprio nem sempre significa uma piora das condições de vida da família. Nesse sentido, a fala abaixo indica uma conotação positiva ao mudarem para uma casa cedida pela filha.

*Essa casa que eu estou morando, ela que comprou* [filha mais velha do primeiro casamento]*, porque na época da primeira entrevista* [2012], *eu morava na comunidade. Era bem difícil ver o esgoto e o lixo na porta de casa.* (M7, 46 anos, mãe de adulto jovem de 19 anos que fazia uso de traqueostomia e gastrostomia)

O número de famílias que paga aluguel manteve-se próximo nos últimos dez anos. Todavia, os relatos indicam o quanto esse valor mensal é oneroso:

*Ajudaria muito se nós tivéssemos casa própria, porque é duro: parece que você paga, você fecha o olho e no outro dia já tem o aluguel. Mas mesmo que a gente não tenha casa própria, nós temos estabilidade dentro de casa. Compra aqui, compra ali, aperta aqui, aperta ali, mas vai indo*. (M2, 46 anos, mãe de adulto jovem de 22 anos que realizava cateterismo vesical intermitente)

*Agora eu arrumei essa casinha mais em conta. Tá dando para ir.* (M9, mãe de um adolescente de 14 anos que fazia uso de traqueostomia e gastrostomia)

Ao comparar a renda familiar de 2012 e 2022, observa-se que não houve diferença estatisticamente significante entre as variáveis “renda familiar em reais” e “renda familiar em salário mínimo”, conforme apresentado na Tabela 2. Ademais, o percentual de famílias contempladas com benefícios governamentais também manteve-se similar, sem diferença estatística. Tal resultado indica que as famílias estudadas, em geral, mantiveram-se na classe D (um a três salários mínimos) e E (até um salário mínimo), de acordo com a classificação do IBGE.

**Tabela 2:** Distribuição das variáveis “renda familiar” e “benefício governamental” segundo o ano (2012 e 2022), Ribeirão Preto, São Paulo, Brasil, 2024

	2012	2022	Valor de *p*
Renda familiar (reais)			0,33^**^
Média (DP)	2.817 (2.104)	3.151 (2.464)	
Mediana (Mín.-Máx.)	2.311 (513-10.273)	2.500 (1.280-12.000)	
Renda familiar (salário mínimo)			0,91^**^
Média (DP)	2,64 (1,97)	2,6 (2,03)	
Mediana (Mín.-Máx.)	2,17 (0,48-9,63)	2,06 (1,06-9,9)	
Benefício governamental			0,32^*^
Sim	15 (65,22%)	18 (75%)	
Não	8 (34,78%)	6 (25%)	
Missings	1	0	

Mín. - mínimo; Máx. - máximo; DP - desvio-padrão; Missings - dados ausentes;

* Teste de Wilcoxon considerando amostras pareadas;

** Teste de McNemar.

Uma possível hipótese para explicar a persistência de menor poder aquisitivo dessas famílias no decorrer dos anos é o constante desafio dessas mulheres de serem incorporadas ao mercado de trabalho formal. A necessidade de observação e cuidado contínuo, em geral, manteve-se mesmo com os filhos adolescentes ou adultos jovens.

*Eu sempre tive vontade de trabalhar registrado, nunca pude. Como que eu vou trabalhar registrado desse jeito? Você acha que o patrão aceitaria eu vou falar para ele “ó, tenho uma filha especial, tenho que correr lá em casa. Ó, aconteceu isso, tem que correr”? Porque vira e mexe tem.* (M2, 46 anos, mãe de adulto jovem de 22 anos que realizava cateterismo vesical intermitente)

*Eu nunca consegui trabalhar depois que ele nasceu. Eu era cozinheira e babá. Eu mais viajava do que ficava em Ribeirão. Eu trabalhava com uma família, e eles viajavam muito para Minas, e eu ia muito com eles. Trabalhei lá oito anos. Aí eu casei, engravidei e saí* […]. *Meu trabalho hoje é meu filho.* (M6, 50 anos, mãe de adolescente de 15 anos que fazia uso de traqueostomia e gastrostomia)

*Eu trabalhava, mas parei porque já não dava mais para ficar. É porque eu não dormia. À noite, ele tinha crises, ele ficava acordado, e eu tinha que ficar acordada para dar atenção a ele, para que não acontecesse nada de grave.* (M10, 52 anos, mãe de adolescente de 17 anos que fazia uso de traqueostomia e gastrostomia)

*Eu doei minha vida para cuidar dele. A partir do momento que eu peguei ele, ele precisava de cuidados 24 horas. Ele ficava no oxigênio, comia por sonda. Até para dormir tinha que ficar atento, tinha medo que algo pudesse acontecer.* (M12, 64 anos, mãe adotiva de adolescente de 15 anos com mobilidade reduzida e uso de derivação ventriculoperitoneal)

Na intenção de ajudar com as despesas domésticas, algumas mães buscaram exercer trabalhos informais.

*Sempre pego alguma coisa para fazer: lingerie para vender, faço unha, pego roupa para passar. Sempre para poder ajudar um pouquinho mais.* (M5, 46 anos, mãe de adolescente de 15 anos que realizava lavagem intestinal e cateterismo vesical intermitente)

*Toda vez que tinha feira, eu pegava minhas coisinhas* [artesanato]*, levava, vendia e comprava o que tinha de comprar para dentro de casa.* (M2, 46 anos, mãe de adulto jovem de 22 anos que realizava cateterismo vesical intermitente)

Além da renda familiar, outras variáveis socioeconômicas consideradas pelo IBGE para classificar a classe social foram investigadas no presente estudo, a saber: número de pessoas que vivem no domicílio, as dimensões da moradia e o acesso à água encanada, rede de esgoto e energia elétrica. Conforme consta na Tabela 3, estimou-se um aumento de 18% no número de moradores após dez anos (p = 0,04).

**Tabela 3:** Distribuição das variáveis “número de moradores”, “número de cômodos” e “presença de água encanada, rede de esgoto e energia elétrica” segundo o ano (2012 e 2022), Ribeirão Preto, São Paulo, Brasil, 2024

	2012	2022	Efeito (IC 95%)	Valor de *p* ^***^
Número de moradores				
Média (DP)	2,78 (1,17)	3,29 (0,69)	1,18 (1,01;1,39)	**0,04**
Mediana (Mín.-Máx.)	2 (1-5)	3 (2-4)		
Número de cômodos			0,88 (0,77;1,01)	0,07
Média (DP)	5,35 (1,56)	4,71 (1,16)		
Mediana (Mín.-Máx.)	6 (2-8)	4,5 (3-8)		
Água encanada, rede de esgoto e energia elétrica				
Sim	23(96%)	24(100%)		
Não	01(4%)	-		

Mín. - mínimo; Máx. - máximo; DP - desvio-padrão;

*** Modelo de regressão binomial negativo com função de ligação logarítmica considerando medidas repetidas.

O aumento no número de moradores pode ser explicado pela junção de pessoas numa mesma residência já que algumas famílias passaram a residir em casas cedidas. Por outro lado, na última década, desafios nas relações conjugais culminaram com a saída do companheiro do lar, como exemplificado pela Mãe 7.

*O pai dela separou de mim, porque ele não dava conta. Ele só queria saber de beber, e era muita briga. Então, assim* […] *para você estar com a pessoa e você nunca poder contar com ele, então é melhor ficar só.* (M7, 46 anos, mãe de adulto jovem de 19 anos que fazia uso de traqueostomia e gastrostomia)

Outra variável socioeconômica analisada foi o tipo de atendimento à saúde recebido. De acordo com a Tabela 4, houve uma redução de 50% entre aqueles que eram atendidos pelo SUS e convênio simultaneamente (atendimento misto), consequentemente ampliou-se o número de famílias que passaram a utilizar exclusivamente o Sistema Único de Saúde (SUS).

**Tabela 4:** Distribuição do tipo de atendimento à saúde segundo o ano (2012 e 2022), Ribeirão Preto, São Paulo, Brasil, 2024

Tipo de atendimento à saúde	2012	2022
SUS	13 (54,2%)	17 (70,8%)
Convênio	1 (4,2%)	2 (8,4%)
Misto	10 (41,6%)	5 (20,8%)

Tal cenário apresentou-se desfavorável na medida em que a insatisfação com os serviços públicos, em especial, com os serviços da Atenção Primária à Saúde, foi destacada nos relatos qualitativos. Para tanto, a assistência ofertada pelo convênio caracterizou-se como complementar, conforme a fala da Mãe 6.

*Porque o meu filho é invisível. Já fui no postinho umas 300 milhões de vezes. O meu filho é invisível para o SAD* [Serviço de Assistência Domiciliar]*, parece que o meu filho não existe. A gente tem uma médica que vem todo mês. Eu trocava lá no HC* [referindo-se à cânula de traqueostomia]*. Aí, quando entrou a pandemia, a gente ficou com medo de levar, e a gente pediu no convênio para a médica vir todo mês trocar. Ela vem pelo* home care *do convênio.* (M6, 50 anos, mãe de adolescente de 15 anos que fazia uso de traqueostomia e gastrostomia)

## DISCUSSÃO

De acordo com os resultados apresentados, não foi evidenciada diferença estatisticamente significante na renda familiar, tanto em reais quanto em salários mínimos, nos últimos dez anos, sendo que as famílias estudadas permaneceram nas classes sociais mais pobres, conforme classificação do IBGE. Além disso, observou-se um aumento de 18% no número de moradores por residência, o que impactou negativamente o poder aquisitivo das famílias à medida que o valor per capita diminuiu.

Embora o diabetes *mellitus* tipo 1 (DM1) não seja caracterizado como uma condição crônica complexa, esse resultado corrobora o estudo realizado no estado da Paraíba com 81 responsáveis por crianças e adolescentes com DM1, em que 40,7% (n = 33) viviam com renda familiar inferior a um salário mínimo, e 58% (n = 47) residiam em domicílios com três ou quatro pessoas adicionais além da criança ou adolescente^(15)^.

Segundo investigação desenvolvida por pesquisadores da Fundação Oswaldo Cruz (FIOCRUZ) que analisou os desafios para a desospitalização e estabilidade clínica de crianças com condições crônicas complexas de saúde, é essencial abordar as questões sociais. Os autores identificaram que a maioria das famílias tinha renda inferior a um quarto do salário mínimo per capita, sendo o Benefício de Prestação Continuada (BPC) a principal fonte de renda. Esse dado revela uma situação de vulnerabilidade social que desafia o processo de desospitalização^(16)^.

Por outro lado, um estudo norte-americano conduzido com 601 pais de indivíduos com idades entre 0 e 28 anos, em cuidados paliativos, buscou determinar a relação entre dificuldades financeiras e sofrimento psicológico dos pais. Identificou-se que aproximadamente 13,2% (n = 79) relataram rendas familiares inferiores a US$ 20 mil, abaixo da linha de pobreza nacional de US$ 21.960 para uma família de três pessoas^(17)^, percentual inferior ao observado no presente estudo.

Adicionalmente, os autores aplicaram uma medida subjetiva para avaliar a dificuldade financeira, questionando os participantes sobre o nível de dificuldade para pagar contas nos últimos seis meses. Identificou-se que 65% relataram algum grau de dificuldade financeira. Esse achado permitiu a formulação da hipótese de que desafios financeiros podem ocorrer em todos os níveis de renda, sugerindo que a percepção de dificuldade financeira estende-se além do montante de renda^(17)^.

No presente estudo, verificou-se que, entre as 24 famílias participantes, 60,9% residiam em casas próprias em 2012, percentual que caiu para 41,6% em 2022. Em comparação, um estudo canadense que investigou o impacto do cuidado parental em 15 famílias de crianças e jovens com complexidade médica revelou que cerca de 70% possuíam casa própria. Contudo, os participantes também relataram os desafios relacionados aos altos custos de adaptação de moradias e veículos para maior acessibilidade dos filhos^(18)^.

Outro aspecto relevante é o aumento expressivo nos preços de alimentos, aluguéis e outras despesas básicas, resultando em perda do poder de compra da população. Segundo o site do DIEESE - Departamento Intersindical de Estatística e Estudos Socioeconômicos, a cesta básica passou de R$ 304,90 em dezembro de 2012 para R$ 791,29 em dezembro de 2022, um aumento em torno de 50% em 10 anos (como referência, a capital de São Paulo)^(19)^.

De maneira geral, os estudos sobre o impacto financeiro em famílias com membros com condições crônicas de saúde distinguem custos diretos e indiretos. Os custos diretos incluem despesas como aquisição de medicamentos, insumos, pagamento de convênios médicos e transporte para hospitais^(11)^. No presente estudo, verificou-se uma redução nos atendimentos via convênio ao longo dos dez anos, demonstrando outra repercussão socioeconômica negativa. Já os custos indiretos estão associados a mudanças na produtividade e emprego, como a necessidade de redução ou abandono do trabalho pelos cuidadores, acarretando perda de renda^(11)^.

Dessa perspectiva, os resultados reafirmaram o constante desafio enfrentado pelas mães cuidadoras para inserção no mercado de trabalho formal. Essa problemática foi enfatizada em estudo que analisou os impactos da paralisia cerebral na saúde e nas finanças^(20)^. Segundo os pesquisadores, o impacto econômico relacionado à perda de renda dos cuidadores informais (geralmente mães) é significativo uma vez que os custos diretos com assistência médica em crianças com paralisia cerebral de 1 a 4 anos foram de aproximadamente US$ 11.700, em comparação com cerca de US$ 600 para crianças sem a condição.

A dificuldade de inserção no mercado de trabalho, associada à sobrecarga das demandas de cuidado, é recorrentemente destacada na literatura. Em um estudo norte-americano, observou-se que os cuidadores precisaram reorganizar suas rotinas laborais para atender às necessidades dos filhos com condições médicas complexas: 36% deixaram o emprego, 66% mantiveram-se no trabalho com redução da carga horária, e 46,2% evitaram trocar de emprego para não perder o seguro de saúde^(21)^. Em pesquisa nacional realizada com pais de crianças e adolescentes com necessidades especiais de saúde, ficou evidenciado que a rotina exaustiva, somada à falta de apoio e à intensa demanda de cuidados, levou o cuidador principal - geralmente a mãe - a abandonar o trabalho^(22)^.

Em outro estudo, realizado em Hong Kong, sobre o ônus socioeconômico de doenças raras, constatou-se que os custos diretos e indiretos representaram mais de 60% do custo total. Entretanto, a idade mais avançada da pessoa com a doença rara (p < 0,0001) e o maior tempo desde o diagnóstico (p = 0,026) estiveram negativamente associados ao custo total médio anual^(23)^.

Cabe ainda problematizar que a coleta de dados ocorreu entre 2022 e 2023, período que coincidiu com o encerramento da Emergência de Saúde Pública de Importância Internacional (ESPII), oficialmente declarado pela Organização Mundial da Saúde em 5 de maio de 2023. Esse contexto gerou impactos socioeconômicos consideráveis para a população. Segundo um estudo de revisão que investigou os efeitos dos determinantes sociais durante a pandemia de covid-19 na população pediátrica, observou-se aumento da insegurança habitacional e alimentar, além do crescimento global dos índices de pobreza^(24)^. Corroborando esses achados, um estudo qualitativo realizado com 27 pessoas que contraíram covid-19 identificou as mudanças financeiras como um dos principais impactos negativos da pandemia, com destaque para o desemprego, impossibilidade de trabalhar e custos associados ao tratamento da doença - fatores que afetaram mais acentuadamente famílias já em situação de maior vulnerabilidade socioeconômica^(25)^.

Por fim, destaca-se o alinhamento desta investigação com a “Agenda 2030 para o Desenvolvimento Sustentável”^(26)^, na medida em que explorou a inter-relação entre saúde e bem estar, erradicação da pobreza e da fome, bem como redução das desigualdades. Essa abordagem é particularmente relevante, considerando os achados de uma revisão de escopo que teve por objetivo identificar e analisar a produção científica sobre a relação entre a enfermagem e os Objetivos de Desenvolvimento Sustentável (ODS). Os resultados dessa revisão indicaram que a prática da enfermagem ainda mantém um foco predominantemente restrito ao ODS 3 - Saúde e Bem-Estar, por ser considerado o mais diretamente associado ao exercício profissional da enfermagem^(27)^. Nesse sentido, pesquisadores(as) têm argumentado que a saúde deve ser abordada por meio de uma “lente ampliada”, que extrapole os modelos tradicionais centrados no indivíduo e na doença, incorporando determinantes sociais, econômicos e ambientais^(28,29)^.

### Limitações do estudo

Considera-se como uma limitação deste estudo o número reduzido de participantes, correspondendo a apenas 20% daqueles que integraram a pesquisa realizada há dez anos. No entanto, a amostra restrita não compromete a validade dos achados e reforça a relevância da realização de novos estudos de natureza longitudinal, com a inclusão de um número mais amplo de famílias. Por fim, recomenda-se que pesquisas futuras considerem, sempre que possível, a inclusão da perspectiva de adolescentes e adultos jovens com condições crônicas complexas.

### Contribuições para área

Este estudo contribui para o avanço do conhecimento em enfermagem pelo seu ineditismo ao integrar resultados quantitativos e qualitativos para explorar as repercussões socioeconômicas em mães de adolescentes e adultos jovens com condições crônicas complexas. Ainda, os achados do presente estudo apresentam potencial para subsidiar o planejamento de ações intersetoriais que busquem promover uma assistência integral, especialmente às famílias em situação de pobreza.

Nesse sentido, recomenda-se o desenvolvimento e a implementação de políticas públicas que ampliem o suporte às famílias cuidadoras. Tais políticas incluem o acesso a programas de transferência de renda vinculados ao cuidado de pessoas com condições crônicas, a prioridade em programas habitacionais, a oferta de oportunidades de capacitação profissional e a flexibilização das condições de trabalho, incluindo a concessão de licenças específicas para o acompanhamento de tratamentos contínuos ou internações frequentes dos filhos.

## CONCLUSÕES

De maneira geral, o estudo identificou que a renda familiar permaneceu semelhante ao longo dos anos, todavia o consumo e o acesso a bens como casa própria e convênio médico reduziram. Os depoimentos reforçaram os achados quantitativos, sobretudo ao retratarem os desafios do trabalho formal, as estratégias para complementar a renda mensal e o custo elevado do aluguel.

Por fim, esta pesquisa alinha-se às prioridades políticas e contribui com a produção de evidências científicas capazes de orientar gestores e profissionais das áreas da saúde e da proteção social na formulação e implementação de ações voltadas à redução das iniquidades em saúde e à melhoria das condições de vida de adolescentes e jovens adultos com condições crônicas complexas, bem como de suas famílias.

## Data Availability

Os dados da pesquisa estão disponíveis no corpo do artigo.
